# Neighborhood Deprivation and Association With Neonatal Intensive Care Unit Mortality and Morbidity for Extremely Premature Infants

**DOI:** 10.1001/jamanetworkopen.2023.11761

**Published:** 2023-05-11

**Authors:** Brynne A. Sullivan, Ayush Doshi, Pavel Chernyavskiy, Ameena Husain, Alexandra Binai, Rakesh Sahni, Karen D. Fairchild, J. Randall Moorman, Colm P. Travers, Zachary A. Vesoulis

**Affiliations:** 1 Division of Neonatology, Department of Pediatrics, University of Virginia School of Medicine, Charlottesville; 2 currently a medical student at University of Virginia School of Medicine, Charlottesville; 3 Department of Public Health Sciences, University of Virginia School of Medicine, Charlottesville; 4 Division of Newborn Medicine, Department of Pediatrics, Washington University in St Louis, St Louis, Missouri; 5 Division of Neonatology, Department of Pediatrics, Columbia University Vagelos College of Physicians and Surgeons, New York, New York; 6 Division of Cardiology, Department of Medicine, University of Virginia School of Medicine, Charlottesville; 7 Division of Neonatology, Department of Pediatrics, University of Alabama at Birmingham

## Abstract

**Importance:**

Socioeconomic status affects pregnancy and neurodevelopment, but its association with hospital outcomes among premature infants is unknown. The Area Deprivation Index (ADI) is a validated measure of neighborhood disadvantage that uses US Census Bureau data on income, educational level, employment, and housing quality.

**Objective:**

To determine whether ADI is associated with neonatal intensive care unit (NICU) mortality and morbidity in extremely premature infants.

**Design, Setting, and Participants:**

This retrospective cohort study was performed at 4 level IV NICUs in the US Northeast, Mid-Atlantic, Midwest, and South regions. Non-Hispanic White and Black infants with gestational age of less than 29 weeks and born between January 1, 2012, and December 31, 2020, were included in the analysis. Addresses were converted to census blocks, identified by Federal Information Processing Series codes, to link residences to national ADI percentiles.

**Exposures:**

ADI, race, birth weight, sex, and outborn status.

**Main Outcomes and Measures:**

In the primary outcome, the association between ADI and NICU mortality was analyzed using bayesian logistic regression adjusted for race, birth weight, outborn status, and sex. Risk factors were considered significant if the 95% credible intervals excluded zero. In the secondary outcome, the association between ADI and NICU morbidities, including late-onset sepsis, necrotizing enterocolitis (NEC), and severe intraventricular hemorrhage (IVH), were also analyzed.

**Results:**

A total of 2765 infants with a mean (SD) gestational age of 25.6 (1.7) weeks and mean (SD) birth weight of 805 (241) g were included in the analysis. Of these, 1391 (50.3%) were boys, 1325 (47.9%) reported Black maternal race, 498 (18.0%) died before NICU discharge, 692 (25.0%) developed sepsis or NEC, and 353 (12.8%) had severe IVH. In univariate analysis, higher median ADI was found among Black compared with White infants (77 [IQR, 45-93] vs 57 [IQR, 32-77]; *P* < .001), those who died before NICU discharge vs survived (71 [IQR, 45-89] vs 64 [IQR, 36-86]), those with late-onset sepsis or NEC vs those without (68 [IQR, 41-88] vs 64 [IQR, 35-86]), and those with severe IVH vs those without (69 [IQR, 44-90] vs 64 [IQR, 36-86]). In a multivariable bayesian logistic regression model, lower birth weight, higher ADI, and male sex were risk factors for mortality (95% credible intervals excluded zero), while Black race and outborn status were not. The ADI was also identified as a risk factor for sepsis or NEC and severe IVH.

**Conclusions and Relevance:**

The findings of this cohort study of extremely preterm infants admitted to 4 NICUs in different US geographic regions suggest that ADI was a risk factor for mortality and morbidity after adjusting for multiple covariates.

## Key Points

**Question:**

Is socioeconomic deprivation at the neighborhood level, measured by an Area Deprivation Index, an independent risk factor for neonatal intensive care unit (NICU) mortality and morbidity among extremely premature infants?

**Findings:**

In a cohort study of 2765 extremely premature infants (gestational age <29 weeks) admitted to 4 level IV NICUs in different US regions, national Area Deprivation Index percentile was associated with the risk of NICU mortality and morbidities after adjusting for multiple covariates.

**Meaning:**

These findings have implications for public health measures, which should target areas of neighborhood deprivation to improve prenatal care with the aim of improving NICU outcomes.

## Teaser

This cohort study investigates the association of the Area Deprivation Index with in-hospital mortality of extremely premature infants and 3 key morbidities, including late-onset sepsis, necrotizing enterocolitis, and severe intraventricular hemorrhage.

## Introduction

Socioeconomic disadvantage impacts maternal health and access to care, affecting the preterm delivery rate^1,2,3^ and infant mortality.^4^ Once premature infants leave the neonatal intensive care unit (NICU), socioeconomic deprivation in the home environment adversely impacts neurodevelopmental outcomes.^5,6^ Evidence suggests that socioeconomic disparity increases adverse outcomes of extremely preterm infants during the NICU course,^7^ but the interaction among deprivation, race, and other risk factors is not well established. Racial disparities confound the effects of socioeconomic status, with families of racial minority groups bearing a higher burden of adverse health outcomes and lower quality of health care,^8,9,10,11^ even among those with higher socioeconomic status and educational levels.^12,13,14^ In addition, due in part to a history of housing discrimination and structural racism in the US, members of racial minority groups more often live in socioeconomically disadvantaged communities.^15^ These complex interactions between race and socioeconomic status necessitate consideration when evaluating maternal and infant outcomes.

The Area Deprivation Index (ADI) is a metric that measures area disadvantage, determined by 17 census variables, including measures of poverty, educational level, housing, and employment status. Singh and Soahpush^16^ first described this metric in relation to disparities in life expectancy that correlated with area deprivation. Kind et al^17^ adapted, updated, and validated the ADI at the neighborhood level using block groups, the smallest geographical unit used by the US Census Bureau. In the most recent iteration, ADI has been normalized at the national level to generate percentiles that can be easily compared, with higher values indicating greater disadvantage. National ADI percentiles and state ADI deciles using 2018 US Census Bureau data are published online in an interactive map.^18,19^

Associations between ADI and health outcomes have been investigated in many populations, including adult patients with Alzheimer disease,^20^ cardiovascular disease,^21^ and in-hospital COVID-19 mortality.^22^ The ADI has also been used to investigate disparity in neonatal health outcomes, such as the rates of exclusive breastfeeding,^23^ neurobehavioral differences and brain structure in term infants,^24,25^ respiratory morbidity after NICU discharge in infants with bronchopulmonary dysplasia,^26^ and length of stay in infants with neonatal opioid withdrawal syndrome.^27^

In this project, we investigated the association of the ADI, a granular, comprehensive, geospatially linked measure of deprivation, and demographic characteristics with in-hospital mortality of extremely premature infants and 3 key morbidities (late-onset sepsis, necrotizing enterocolitis [NEC], and severe intraventricular hemorrhage [IVH]). We hypothesized that maternal residence in areas with greater deprivation is associated with increased NICU mortality and morbidity risk. Our study includes 8 years of data from 4 regional referral NICUs located in 4 US regions: Midwest, Northeast, Mid-Atlantic, and South.

## Methods

### Study Design and Patient Population

We conducted a multicenter, retrospective cohort study of non-Hispanic Black and White premature infants born at a gestational age of less than 29 weeks and admitted to 1 of 4 academic level IV NICUs—University of Virginia in Charlottesville; University of Alabama at Birmingham; Washington University in St Louis, St Louis, Missouri; and Columbia University, New York City, New York—between January 1, 2012, and December 31, 2020. We followed the Strengthening the Reporting of Observational Studies in Epidemiology (STROBE) reporting guideline. Maternal self-reported race was used to determine the infants’ race. Hispanic infants were excluded because this ethnicity category was not specified in the earlier years of the study, and they accounted for relatively few patients (5%-10%) at all but 1 NICU, at Columbia University. We also excluded Asian infants and other (eg, American Indian and Alaska Native) race categories with very few patients. We excluded infants with missing outcomes data from the analysis. Each institution’s institutional review board reviewed and approved the study under a waiver of informed consent.

### Address Geocoding and ADI Data Process

All geographic locations in the US are defined using a Federal Information Processing System code. The degree of granularity of this code is determined by the number of digits, with a maximum of 15 digits to define an individual census block. Neighborhood Atlas ADI data were calculated at the US Census block group level, a statistical division of geographic areas containing between 600 and 3000 people. A block group is a 12-digit number: 2 digits represent the state, 3 digits represent the county, 6 digits represent the census tract, and 1 digit represents the block group.^28^ Some address types such as PO boxes and rural routes do not have corresponding ADI values, and therefore patients with these addresses were excluded from the analysis.

Look up for the patient address listed in the birth encounter was performed in a semiautomated fashion for ADI using a 4-step processing pipeline written in Python, version 3.6 (Python.org) (eMethods in Supplement 1). Once converted, centers discarded address data, and only ADI values were shared for analysis. This way, patient addresses remained local and protected, and shared data were deidentified.

### Clinical and Outcome Data

Clinical data were extracted from unit databases or electronic health records, including demographic characteristics, perinatal variables, and outcomes. The primary outcome was mortality before NICU discharge, and secondary outcomes included late-onset sepsis or NEC and severe IVH. Late-onset sepsis was defined as positive blood culture findings after 3 days of age that was treated with antibiotics for at least 5 days (or less if the infant died during treatment); NEC, as radiographic evidence of pneumatosis, pneumoperitoneum, or portal venous gas plus clinical signs and symptoms of sepsis according to the modified Bell staging criteria for stage 2 or higher^29^; and severe IVH, as grades III to IV IVH identified on cranial ultrasonography according to the Papile classification system.^30^

### Statistical Analysis

We performed univariate comparisons among ADI, demographic characteristics, and primary and secondary outcomes using Wilcoxon rank sum or χ^2^ tests. We analyzed the multivariable association between ADI and NICU outcomes using bayesian logistic regression adjusted for race, birth weight, outborn status, and sex. For each variable included in the multivariable models, we also performed univariate bayesian logistic regression analysis with primary and secondary outcomes. For computational efficiency, quantitative variables were centered and scaled before analysis. Two-sided *P* < .05 indicated statistical significance.

In contrast with frequentist models, bayesian models provide more information about parameters through posterior distributions,^31^ more correctly quantify the uncertainty in model parameters,^32^ and are often able to estimate models that may otherwise fail.^33^ We used weakly informative priors for all parameters. We considered a risk factor significant if the posterior 95% credible intervals (CrI) did not include zero and took the posterior median as the summary of the posterior in all regression analyses. We compared models with interaction terms to the baseline model and considered an interaction significant if the information criterion value was lower than that of the baseline model.

## Results

### Patient Characteristics

We analyzed ADI and clinical data from 2765 extremely premature infants admitted during the study period. The mean (SD) gestational age was 25.6 (1.7) weeks; birth weight, 805 (241) g. In terms of sex and race, 1391 infants (50.3%) were boys and 1374 (49.7%) were girls, and 1325 (47.9%) reported Black maternal race and 1440 (52.1%) reported White maternal race. A total of 498 infants (18.0%) died before NICU discharge, 692 (25.0%) were diagnosed with sepsis or NEC, and 353 (12.8%) developed severe IVH. Inborn status ranged from 261 of 405 infants (64.4%) at Washington University in St Louis to 396 of 504 (78.6%) at Columbia University. Table 1 summarizes cohort characteristics by center. Missing data occurred in 387 infants (14.0%), which required excluding them from multivariable modeling. Of these infants, 235 were missing data on birth hospital to determine inborn or outborn status, 83 were missing data on IVH grade, and 69 were missing data on whether or not sepsis or NEC was diagnosed during the NICU course. Only 37 infants (1.3%) were excluded due to their address being missing or not linked to a block group with an ADI percentile (PO boxes, rural routes).

**Table 1.** Patient Characteristics by NICU

Characteristic	NICU^a^			
	UVA (n = 508)	WUSTL (n = 405)	CU (n = 504)	UAB (n = 1348)
Median ADI national percentile (IQR)	50 (36-66)	75 (53-91)	16 (7-27)	81 (64-92)
Gestational age, median (IQR), wk	26 (24-27)	26 (24-27)	26 (24-27)	26 (24-27)
Birth weight, median (IQR), g	815 (650-991)	840 (650-1040)	760 (624-965)	740 (587-950)
Sex				
Female	235 (46.3)	192 (47.4)	251 (49.8)	696 (51.6)
Male	273 (53.7)	213 (52.6)	253 (50.2)	652 (48.4)
Race				
Black	137 (27.0)	175 (43.2)	252 (50.0)	761 (56.5)
White	371 (73.0)	230 (56.8)	252 (50.0)	587 (43.5)
Mortality	68 (13.4)	72 (17.8)	81 (16.1)	277 (20.5)
Inborn	361 (71.1)	261 (64.4)	396 (78.6)	963 (71.4)
Sepsis or NEC^b^	123 (24.2)	90 (22.2)	91 (18.1)	388 (28.8)
Severe IVH^c^	71 (14.0)	94 (23.2)	26 (5.0)	162 (12.0)

Abbreviations: ADI, Area Deprivation Index; CU, Columbia University; IVH, intraventricular hemorrhage; NEC, necrotizing enterocolitis; UAB, University of Alabama at Birmingham; UVA, University of Virginia; WUSTL, Washington University in St Louis.

^a^ Unless otherwise indicated, data are expressed as No. (%) of infants.

^b^ Indicates Bell stage 2 or 3.

^c^ Indicates grades III to IV.

### Univariate Analysis

**Figure 1.**
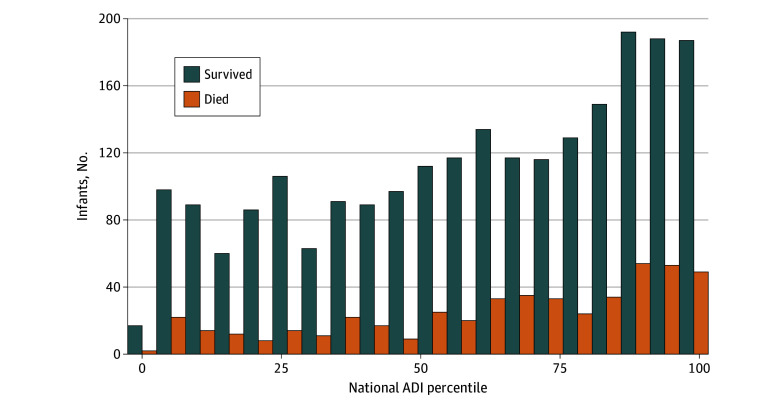
National Area Deprivation Index (ADI) and Neonatal Intensive Care Unit (NICU) Mortality The bar graph shows the number of extremely premature infants in each ADI percentile who died before NICU discharge or survived. Higher ADI indicates greater disadvantage.

In pooled data across the 4 centers, the distribution of ADI was skewed, with 1770 infants (64.0%) born to families residing in areas of higher deprivation, or ADI above the fiftieth national percentile. Mortality was similarly skewed, with more deaths among the infants with higher ADI (Figure 1) and higher ADI among infants who died before discharge compared with survivors (71 [IQR, 45-89] vs 64 [IQR, 36-86]; *P* < .001). Among the 1777 infants (64.0%) with ADI above the national median, mortality was 20.0% (n = 355) compared with 14.0% (n = 138) in the 988 infants with ADI at or below the national median (*P* < .001). Higher ADI was also associated with self-reported Black maternal race (77 [IQR, 45-93] vs 57 [IQR, 32-77]), inborn status (63 [IQR, 35-86] vs 53 [IQR, 29-76]), diagnosis with sepsis or NEC (68 [IQR, 41-88] vs 64 [IQR, 35-86]), and severe IVH (69 [IQR, 44-90] vs 64 [IQR, 36-86]) (Table 2). Families of Black infants lived in block groups with higher ADI than White infants (median ADI, 77 [IQR, 45-93] vs 57 [IQR, 32-77]; *P* < .001) (eFigure 1 in Supplement 1). Black infants had higher mortality (265 of 1325 [20.0%] vs 233 of 1440 [16.2%]; *P* = .01) and lower mean (SD) birth weight (785 [239] vs 824 [241] g; *P* < .001) than White infants. We found no correlation between birth weight and ADI (*r* = −0.05). Significant intercenter variation in ADI existed, as shown eFigure 2 in Supplement 1.

**Table 2.** Covariates and ADI Distributions

Binary subgroup comparisons	Median national ADI percentile (IQR)	
	Group A	Group B
Died (A) vs survived (B)^a^	71 (45-89)	64 (36-86)
Black (A) vs White (B) race^a^	77 (45-93)	57 (32-77)
Girls (A) vs boys (B)	68 (38-87)	64 (37-86)
Inborn (A) vs outborn (B)^a^	63 (35-86)	53 (29-76)
Sepsis or NEC (A) vs neither (B)^a^	68 (41-88)	64 (35-86)
Severe IVH (A) vs mild or no IVH (B)^a^	69 (44-90)	64 (36-86)

Abbreviations: ADI, Area Deprivation Index; IVH, intraventricular hemorrhage; NEC, necrotizing enterocolitis.

^a^ *P* < .01 by Wilcoxon rank sum tests.

Comparing male and female infants, we found no statistically significant difference in ADI (median, 64 [IQR, 37-86] and 68 [IQR, 38-87], respectively), race (eg, 741 of 1391 [53.3%] and 699 of 1374 [50.9%] White, respectively), or the proportion of outborn infants (223 of 1205 [16.0%] vs 185 of 1184 [13.5%], respectively). As expected, the mean (SD) birth weight was slightly higher for male than female infants (836 [243] vs 774 [235] g; *P* < .001). While mortality was not significantly higher for male (159 of 1391 [18.6%]) than female infants (239 of 1374 [17.4%]) overall, White male infants had higher mortality than White female infants (128 of 741 [17.3%] vs 105 of 699 [15.0%]; *P* = .006), while mortality among Black infants did not differ by sex (131 of 650 [20.2%] vs 134 of 675 [19.9%]).

While most patients (1981 [71.6%]) were admitted following delivery at the same hospital, outborn infants had lower ADI (Table 2) and lower mortality (59 of 408 [14.5%] vs 375 of 1981 [18.9%]; *P* = .04), and a higher proportion were White race (262 of 408 [64.2%] vs 1002 of 1981 [50.6%]; *P* < .001). Infants transferred to the study NICU after delivery at an outside hospital also had a higher rate of severe IVH (outborn, 91 of 408 [22.3%]; inborn, 217 of 1981 [12.2%]; *P* < .001).

**Figure 2.**
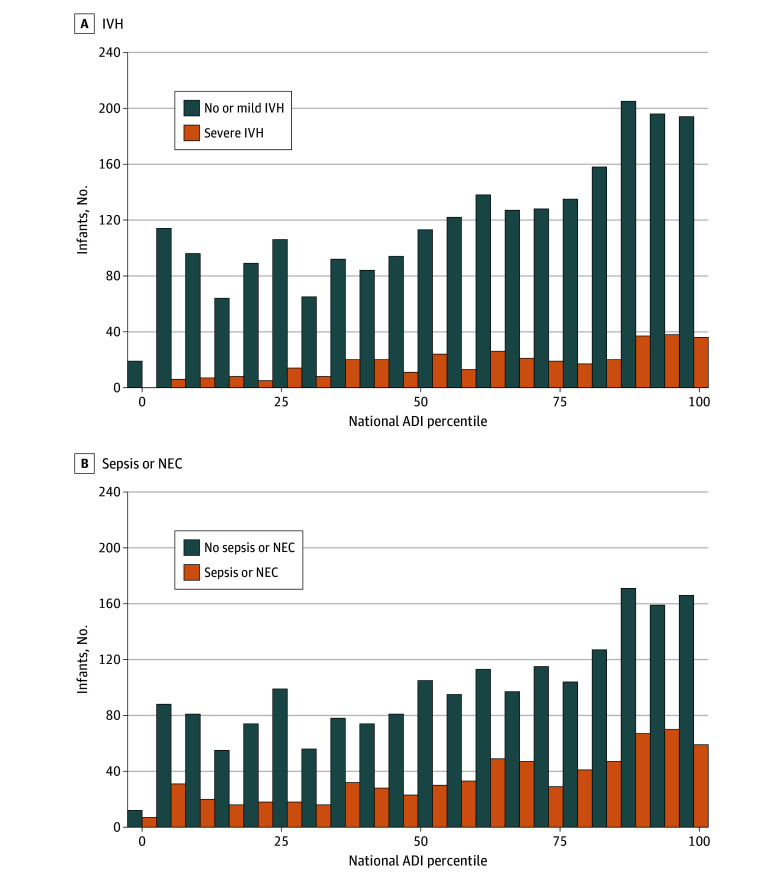
National Area Deprivation Index (ADI) Percentiles and Neonatal Intensive Care Unit (NICU) Morbidity The bar graph shows the number of infants in each ADI percentile grouped by whether or not infants had no or mild intraventricular hemorrhage (IVH; grades I-II) vs severe IVH (grades III-IV) (A) and 1 or more episodes of confirmed sepsis or necrotizing enterocolitis (NEC) (B).

Infants with sepsis or NEC, severe IVH, or both had higher ADI than those without (Table 2 and Figure 2). Compared with 18.0% overall mortality, 167 (24.1%) of infants with sepsis or NEC (*P* < .001) and 124 (35.1%) of those with severe IVH (*P* < .001) died. Compared with mean (SD) birth weight of 805 (241) g in the overall cohort, those diagnosed with sepsis or NEC had lower mean (SD) birth weight (731 [209] g; *P* < .001) as did those with severe IVH (746 [217] g; *P* < .001). Sepsis and NEC and severe IVH rates did not differ among infants grouped by sex or race.

In addition to comparing variable distributions among binary subgroups, we performed univariate bayesian logistic regression to estimate the association between each variable used in multivariable modeling and the primary and secondary outcomes. Table 3 summarizes the univariate and multivariable modeling results for each outcome.

**Table 3.** Univariate and Multivariable Model Results

Outcome	Risk factor	Posterior estimate (95% CrI)^a^	
		Univariate	Multivariable
Mortality	Birth weight	−1.18 (−1.31 to −1.05)^b^	−1.20 (−1.35 to −1.06)^b^
	Male sex	0.08 (−0.11 to 0.29)	0.35 (0.13 to 0.57)^b^
	ADI percentile	0.18 (0.08 to 0.29)^b^	0.14 (0.02 to 0.26)^b^
	Outborn	−0.32 (−0.62 to −0.04)^b^	−0.27 (−0.58 to 0.05)
	White race	−0.26 (−0.45 to −0.06)^b^	−0.03 (−0.26 to 0.20)
Sepsis or NEC	Birth weight	−0.45 (−0.55 to −0.36)^b^	−0.44 (−0.55 to −0.33)^b^
	Male sex	0.08 (−0.12 to 0.28)	0.22 (0.02 to 0.41)^b^
	ADI percentile	0.11 (0.02 to 0.20)^b^	0.13 (0.04 to 0.23)^b^
	Outborn	−0.46 (−0.71 to −0.20)^b^	−0.39 (−0.68 to −0.12)^b^
	White race	−0.14 (−0.31 to 0.04)	−0.06 (−0.25 to 0.13)
Severe IVH	Birth weight	−0.32 (−0.44 to −0.21)	−0.33 (−0.46 to −0.19)^b^
	Male sex	0.20 (0.09 to 0.32)^b^	0.12 (−0.11 to 0.36)
	ADI percentile	0.17 (−0.05 to 0.39)	0.27 (0.14 to 0.40)^b^
	Outborn	0.84 (0.14 to 0.56)^b^	0.93 (0.65 to 1.21)^b^
	White race	0.12 (−0.21 to 0.25)	0.11 (−0.14 to 0.37)

Abbreviations: ADI, Area Deprivation Index; CrI, credible interval; IVH, intraventricular hemorrhage; NEC, necrotizing enterocolitis.

^a^ Calculated as bayesian regression models estimating the 3 outcomes. Birth weight and ADI percentile were centered and scaled.

^b^ Variables were considered statistically significant if the 95% CrI did not include zero.

### Results of Multivariable Analysis

We examined the association between ADI and NICU mortality while adjusting for the covariates identified in univariate analyses: birth weight, sex, race, and outborn status. Based on posterior median estimates, birth weight was the strongest risk factor for mortality (posterior estimate, −1.20 [95% CrI, −1.35 to −1.06]), but national ADI percentile (posterior estimate, 0.14 [95% CrI, 0.02-0.26]) and male sex (posterior estimate, 0.35 [95% CrI, 0.13-0.57]) were also associated with mortality (Table 3). Neither race nor the interaction between race and ADI was significant, and the interaction term did not improve the Watanabe-Akaike information criteria^34^ of the model.

Birth weight and ADI were also associated with NICU morbidity in multivariable models (Table 3). Because we scaled and centered continuous variables, we were able to compare the size of the estimates among variables included in each model. By treating ADI and birth weight values as *z* scores, we report the effect of changing either variable by 1 SD, rather than by 1 percentile (ADI) or 1 g (birth weight). Therefore, in the model for mortality, we interpreted the effect of decreasing birth weight by 1 SD to be 9 times that of increasing ADI by 1 SD. In contrast, in the model for severe IVH, the effects of birth weight and ADI were similar and still less than the effect of outborn status. When modeling sepsis and NEC, the effect of decreasing birth weight was about 3 times that of increasing ADI, and male sex and outborn status had intermediate effects. Race did not significantly affect any of the three outcomes modeled.

## Discussion

Socioeconomic factors play an important role in maternal health, access to prenatal care, rates of preterm birth, and infant morbidity and mortality outside of the NICU.^4,5,35^ While in the NICU, preterm infants are treated according to standardized care guidelines that might be expected to limit the association of socioeconomic deprivation with adverse outcomes. Few studies have evaluated the association of area deprivation with in-hospital outcomes.^36^ In this study, we found that residence in a socioeconomically disadvantaged neighborhood—as measured by the ADI—increases the risk of NICU mortality or morbidity for extremely preterm infants, even after accounting for other known risk factors. This indicates a pervasive and detrimental effect of area deprivation that may not be entirely eliminated by provision of standardized, high-quality NICU care.

The mechanism by which area deprivation affects neonatal outcomes may include the effect of maternal adversity on the developing fetus.^37,38^ Maternal stress and poor access to prenatal care have been linked with adverse birth outcomes.^39,40,41^ Lower maternal socioeconomic status increases the risk of preterm birth,^11,12,42^ and among those born prematurely, it increases the risk of adverse neurodevelopmental outcomes.^43,44^ In contrast with other studies, we did not find an association between ADI and birth weight, likely due to the fact that we limited the analysis to extremely premature infants with a narrow range of birth weights.

We found that the association between higher deprivation and mortality remained in multivariable analysis while the association between Black race and mortality did not. These highly collinear variables make it difficult to estimate the effect of race or deprivation alone. Studies indicate that despite advances over time, racial disparities persist in neonatal outcomes and care practices,^10,45,46^ but these analyses did not account for socioeconomic factors. As racial minority individuals proportionally endure more socioeconomic deprivation, it becomes essential to consider social disparities as a significant mediator of racial disparities. Public health efforts have been made to narrow disparities in antenatal corticosteroid use and cesarean deliveries, but gaps in these practices persist.^46^ Disparities in breastfeeding by race persist,^10^ despite overall improvements in breastfeeding initiation over time, with the lowest rate of breastfeeding at NICU discharge among infants born to non-Hispanic Black mothers.^47^ In our cohort, infants born to Black mothers were exposed to higher levels of deprivation in utero and had lower birth weights. Still, they did not have higher associated morbidity or mortality when accounting for these and other confounding variables. Our results suggest that the higher mortality observed among Black preterm infants compared with White infants may be partly confounded by factors captured in the ADI, including exposure to adverse social and economic conditions.

### Strengths and Limitations

A strength of this study was the inclusion of 4 centers in geographically distinct areas of the US (Northeast, Mid-Atlantic, Midwest, and South) with different distributions of ADI, patient racial compositions, and population densities. Although all centers are level IV referral NICUs, there were differences in the percentage of inborn infants, ranging from 64.4% to 78.6%. Interestingly, the distribution of ADI was skewed in opposite directions for infants admitted to the centers in the Midwest and South compared with the Northeast, while the Mid-Atlantic center had a nearly symmetric ADI distribution (eFigure 1 in Supplement 1). The ADI distributions within each center appear to be representative of their respective regional distributions as displayed on the Neighborhood Atlas ADI map. We did not adjust for center in multivariable modeling, as national percentile ADI data has already been normalized across all geographic regions. Additionally, we did not use state ADI deciles in our analysis since these values do not translate across state lines and would not be appropriate for a pooled sample including data from multiple states.

Limitations of the study include the lack of granularity in the ADI and race data. Although block groups are the most geospatially compact census unit and the ADI can provide a localized overview of the degree of socioeconomic deprivation, its value is opaque. The relative contributions of each ADI component are not available for the most current values, making it infeasible to evaluate driving factors of the association between ADI and NICU morbidity and mortality. In addition, the exposures captured in the ADI may have differential impacts across the heterogeneous locations that we analyzed. For example, in the 2020 US Census data for just the 4 cities in which the study NICUs were located, the land area ranged from 10.24 to 302.64 square miles; population density ranged from 1365 to 29 302 per square mile; median household incomes ranged from $38 832 to $67 046; and the percentage of adults without health insurance ranged from 7.9% to 14.7%. Each of these differences likely plays a significant role in the factors that underlie the ADI calculation.

We classified race into broad groups of Black or White based on the self-reported maternal race and excluded American Indian and Alaska Native, Asian, and Hispanic patients, which may have left important interactions or outcomes undiscovered. Analyzing more refined racial and ethnic subgroups, including more than 1 race when the father’s race differs from the mother’s race, may have found differential associations between ADI and outcomes among more finely delineated racial and ethnic groups. For example, the “Hispanic paradox” refers to the paradoxical finding that Hispanic infant mortality rates lie at or below the rate of Non-Hispanic White infant mortality, despite higher socioeconomic deprivation.^48,49^

Our results generate questions for future studies to evaluate how interventions to reduce area deprivation or improve equity might reduce disparities in NICU outcomes for premature infants. The study’s findings also leave us wanting to know more about the mechanism of the association between ADI and NICU outcomes, but we could not answer such questions with the data available.

## Conclusions

In this cohort study of preterm infants born at a gestational age of less than 29 weeks and admitted to 4 NICUs, ADI was a risk factor for NICU mortality and morbidity after adjusting for multiple covariates. These findings highlight the need for further research on the components of the ADI that drive this association. Specific risk factors may be targets of public health measures to improve prenatal care and infant outcomes for patients from disadvantaged areas.

## Notes

Supplement 1. **eMethods.** ADI Data Processing Pipeline **eFigure 1.** National Ranked Area Deprivation Index (ADI) Percentiles Grouped by Race **eFigure 2.** ADI Histograms Grouped by Mortality (Top Panel) and Race (Bottom Panel) for Infants at Each of the 4 NICUs

Supplement 2.
Data Sharing Statement
