# Errors in Figure

**DOI:** 10.1001/jamanetworkopen.2024.56440

**Published:** 2024-12-23

In the Original Investigation titled “Neighborhood Deprivation and Association With Neonatal Intensive Care Unit Mortality and Morbidity for Extremely Premature Infants,”^1^ published May 11, 2023, the panel titles in Figure 2 were transposed, as were their corresponding descriptions in the caption. This article has been corrected.
