# Supplementary material for: Neighborhood Deprivation and Association With Neonatal Intensive Care Unit Mortality and Morbidity for Extremely Premature Infants
Source: JAMA Netw Open. 2023 May 11;6(5):e2311761. doi: 10.1001/jamanetworkopen.2023.11761 (PMC10176121; doi:10.1001/jamanetworkopen.2023.11761)
Supplement: Supplement 2. — Data Sharing Statement [file jamanetwopen-e2311761-s002.pdf]

Data Sharing Statement

Sullivan. Neighborhood Deprivation and Association With Neonatal Intensive Care Unit Mortality and Morbidity for Extremely Premature Infants. *JAMA Netw Open*. Published May 11, 2023. doi:10.1001/jamanetworkopen.2023.11761

Data
Data available: No

Additional Information
Explanation for why data not available: Data are available from the authors upon reasonable request
